# Voxel-level comparison of arterial spin-labeled perfusion magnetic resonance imaging in adolescents with internet gaming addiction

**DOI:** 10.1186/1744-9081-9-33

**Published:** 2013-08-12

**Authors:** Qi Feng, Xue Chen, Jinhua Sun, Yan Zhou, Yawen Sun, Weina Ding, Yong Zhang, Zhiguo Zhuang, Jianrong Xu, Yasong Du

**Affiliations:** 1 Department of Radiology, Ren Ji Hospital, School of Medicine Shanghai Jiao Tong University , Shanghai 200127, P.R. China; 2 Department of Child & Adolescent Psychiatry Shanghai Mental Health Center, Shanghai Jiao Tong University, Shanghai 200030, P.R. China; 3 Ge Applied Science Laboratory, GE Healthcare, Shanghai, P.R. China

**Keywords:** Internet addiction, Internet gaming addiction, Arterial spin-labeling perfusion, fMRI, Cerebral blood flow

## Abstract

**Background:**

Although recent studies have clearly demonstrated functional and structural abnormalities in adolescents with internet gaming addiction (IGA), less is known about how IGA affects perfusion in the human brain. We used pseudocontinuous arterial spin-labeling (ASL) perfusion functional magnetic resonance imaging (fMRI) to measure the effects of IGA on resting brain functions by comparing resting cerebral blood flow in adolescents with IGA and normal subjects.

**Methods:**

Fifteen adolescents with IGA and 18 matched normal adolescents underwent structural and perfusion fMRI in the resting state. Direct subtraction, voxel-wise general linear modeling was performed to compare resting cerebral blood flow (CBF) between the 2 groups. Correlations were calculated between the mean CBF value in all clusters that survived AlphaSim correction and the Chen Internet Addiction Scale (CIAS) scores, Barratt Impulsiveness Scale-11 (BIS-11) scores, or hours of Internet use per week (hours) in the 15 subjects with IGA.

**Results:**

Compared with control subjects, adolescents with IGA showed significantly higher global CBF in the left inferior temporal lobe/fusiform gyrus, left parahippocampal gyrus/amygdala, right medial frontal lobe/anterior cingulate cortex, left insula, right insula, right middle temporal gyrus, right precentral gyrus, left supplementary motor area, left cingulate gyrus, and right inferior parietal lobe. Lower CBF was found in the left middle temporal gyrus, left middle occipital gyrus, and right cingulate gyrus. There were no significant correlations between mean CBF values in all clusters that survived AlphaSim correction and CIAS or BIS-11 scores or hours of Internet use per week.

**Conclusions:**

In this study, we used ASL perfusion fMRI and noninvasively quantified resting CBF to demonstrate that IGA alters the CBF distribution in the adolescent brain. The results support the hypothesis that IGA is a behavioral addiction that may share similar neurobiological abnormalities with other addictive disorders.

## Introduction

Since Kimberly Young (1996) first proposed the idea that problematic computer use meets the criteria for addiction, internet gaming addiction (IGA) has been extensively studied and is currently included in Section 3, the research appendix, of the Diagnostic and Statistical Manual version 5 (DSM-V) [1]. Both China and South Korea have identified IGA as a significant public health threat, and both countries support education, research, and treatment [2]. Although IGA can be considered an impulse control disorder (not otherwise specified), there is a growing consensus that this constellation of symptoms is an addiction [3]. The American Society of Addiction Medicine (ASAM) recently released a new definition of addiction as a chronic brain disorder, and officially proposed for the first time that addiction is not limited to substance abuse [4]. All addictions, whether chemical or behavioral, share certain characteristics. These include salience, compulsive use (loss of control), mood modification and the alleviation of distress, tolerance and withdrawal, and the continuation despite negative consequences. Possibly because diagnostic criteria and assessment questionnaires vary between countries, and because studies often use highly selective samples from online surveys, there is considerable variance in the reported prevalence of IGA (between 0.3% and 38%) [5,6].

Addictions activate a combination of sites in the brain associated with pleasure, known collectively as the reward center of the brain [7,8]. Activation of this center involves increased dopamine release, along with opiates and other neurochemicals. Over time, relevant receptors may be affected. This can engender tolerance, which may manifest as the need for higher levels of stimulation of the reward center to produce a similar “high,” and eventually to engaging in characteristic behaviors to avoid withdrawal. Internet use may also lead specifically to dopamine release in the nucleus accumbens [8], one of the reward structures of the brain specifically involved in other addictions [9].

Recent advances in brain imaging and cognitive neuroscience have led to growing interest in integrating neuroimaging methods and neurocognitive frameworks into the study of IGA, the most common type of internet addiction disorder [10-15]. Using imaging techniques such as functional or structural magnetic resonance imaging (MRI) and diffusion tensor MRI, research has suggested that IGA shares psychological and neural mechanisms with drug addiction [10-15]. However, to date, only two studies have reported perfusion changes in IGA, and those studies used positron emission tomography (PET) [16,17]. ASL perfusion MRI offers absolute quantification of cerebral blood flow (CBF) in units of milliliters of blood per 100 g of tissue per minute, which is normally coupled with neural activity using magnetically labeled arterial blood water as an endogenous tracer [18]. Previous studies have shown excellent reproducibility over long time periods and less between-subject variability with ASL perfusion compared with blood oxygen-dependent level (BOLD) fMRI [19,20]. In the present study, we used arterial spin-labeling (ASL) perfusion functional MRI (fMRI) and measured resting CBF in 2 groups of adolescents, one with and one without IGA. The aims of this study were 1) to investigate the effects of IGA on resting cerebral activation patterns in the adolescent brain noninvasively, 2) to examine whether any alterations are consistent with those seen in the patients with drug addiction, and 3) to determine whether there are any relationships between altered CBF and behavioral and personality measures in subjects with IGA.

## Methods and procedure

### Participant selection

All subjects were recruited from the Department of Child and Adolescent Psychiatry of Shanghai Mental Health Center. They were 14 to 17 years old. We imaged fifteen subjects whose behaviors corresponded with the DSM-IV criteria for IGA according to the modified Diagnostic Questionnaire for Internet Addiction (i.e., the YDQ) criteria by Beard [21]. Eighteen age- and gender-matched healthy individuals with no personal or family history of psychiatric disorders were imaged as the control group. All subjects were right-handed and none of them smoked.

A basic information questionnaire was used to collect demographic information such as gender, age, final year of schooling completed, and hours of Internet use per week. This study was approved by the Ethics Committee of Ren Ji Hospital, School of Medicine Shanghai Jiao Tong University. The participants and their parents or legal guardians were informed of the aims of our study before the MRI examinations were conducted. Full and written informed consent was obtained from the parents or legal guardians of each participant.

All subjects underwent a simple physical examination including blood pressure and heart rate measurements, and were interviewed by a psychiatrist regarding their medical history of nervous, motor, digestive, respiratory, circulation, endocrine, urinary, and reproductive problems. They were then screened for psychiatric disorders with the Mini International Neuropsychiatric Interview for Children and Adolescents (MINI-KID) [22]. The exclusion criteria included a history of substance abuse or dependence, previous hospitalization for psychiatric disorders, or a history of major psychiatric disorders, such as schizophrenia, depression, anxiety disorder, and psychotic episodes. The subjects with IGA were not treated with psychotherapy or any medications.

The diagnostic questionnaire for IGA was adapted from DSM-IV criteria for pathological gambling by Young [23]. The YDQ we used consisted of eight “yes” or “no” questions translated into Chinese. It included the following questions: (1) Do you feel absorbed in the Internet, as indexed by remembering previous online activity or the desire for the next online session? (2) Do you feel satisfied with your Internet use if you increase your amount of online time? (3) Have you failed to control, reduce, or quit Internet use repeatedly? (4) Do you feel nervous, temperamental, depressed, or sensitive when trying to reduce or quit Internet use? (5) Do you stay online longer than originally intended? (6) Have you taken the risk of losing a significant relationship, job, educational opportunity or career opportunity because of the Internet? (7) Have you lied to your family members, therapist, or others to hide the truth of your involvement with the Internet? (8) Do you use the Internet as a way of escaping from problems or of relieving an anxious mood (e.g., feelings of helplessness, guilt, anxiety, or depression)? Young asserted that five or more “yes” responses to these eight questions indicated a dependent user. Later, Beard and Wolf [21] modified the YDQ criteria to state that respondents who answered “yes” to questions 1 through 5 and at least one of the remaining three questions should be classified as suffering from IGA.

### Behavioral and personality assessments

Four questionnaires were used to assess the participants’ behavioral and personality features, namely the Chen Internet Addiction Scale (CIAS) [24], Self-Rating Anxiety Scale (SAS) [25], Self-rating Depression Scale (SDS) [26], and Barratt Impulsiveness Scale-11 (BIS-11) [27]. All questionnaires were initially constructed in English and then translated into Chinese.

### Image acquisition

MRI was conducted using a 3 T MRI scanner (GE Signa HDxt 3 T, USA). A standard head coil with foam padding was used to restrict head motion. During ASL perfusion fMRI, the subjects were instructed to keep their eyes closed, remain motionless, stay awake, and not to think of anything in particular. High-resolution whole brain anatomic images were collected using 3-dimensional magnetization-prepared rapid gradient echo (MPRAGE) with the following parameters: repetition time (TR) = 9.4 ms, echo time (TE) = 4.6 ms, flip angle = 15, slice thickness = 1 mm, gap = 0, field of view (FOV) = 256 mm × 256 mm, matrix = 256 × 256, slices = 155. Pseudocontinuous ASL perfusion images were collected using 3D fast spin-echo acquisition with background suppression, with a labeling duration of 1500 ms and post-labeling delay of 1500 ms, as suggested previously [28]. TR = 4580 ms, TE = 9.8 ms, FOV = 240 × 240 mm, matrix = 128 × 128, flip angle = 155°, slice thickness = 4 mm. CBF maps were calculated from the perfusion-weighted images using a 2-compartment model [29] with a finite labeling duration [4,30], as described previously [31].

### Data analysis

For demographic differences and behavioral and personality measures, two-sample t-tests were used for group comparisons to examine differences between the two groups, and χ^2^-tests were used for gender comparisons. A two-tailed *p*-value of 0.05 was considered statistically significant for all analyses.

For the 3D ASL MRI data, the voxel-based analyses were performed using the statistical parametric mapping version 8 (SPM8) program (Statistical Parametric Mapping 8, Wellcome Department of Imaging Neuroscience, University College, London, UK). For the group analysis, image processing was performed with SPM8 following the method described by Kim et al. [32]. In the coregistration step for each subject, the CBF maps were coregistered to the anatomical three-dimensional (3D) T1-weighted (MPRAGE) image. To correct for partial volume effects in the CBF maps at the voxel-level, the 3D T1-weighted images were segmented to obtain the tissue probabilistic maps of gray matter (GM), white matter (WM), or cerebrospinal fluid (CSF). Using the segmented probabilistic maps, CBF values were corrected according to the following formula: CBF_correct_ = CBF_incorrect_/(GM + 0.4*WM), where CBF correct and CBF incorrect represent CBF after and before partial volume correction, respectively. We assumed that global perfusion of WM is 40% of GM perfusion based on a previous PET study [33-35]. In addition, we only included comparisons of CBF values in voxels composed of more than 50% of gray and whiter matter (i.e., GM + WM > 0.5). This was used to minimize the contribution of CSF and increase the statistical power.

The CBF_correct_ map of each subject was spatially normalized to the EPI template supplied by SPM8. The original voxel size was interpolated into a final voxel size of 2 × 2 × 2 mm^3^. Transformation parameters were then applied to the CBF_correct_ map such that the CBF_correct_ map of each subject was transformed into a standard Montreal Neurological Institute (MNI) space. The normalized CBF_correct_ maps were smoothed for group comparisons with 6 mm full width at half-maximum isotropic Gaussian kernel.

**Figure 1:**
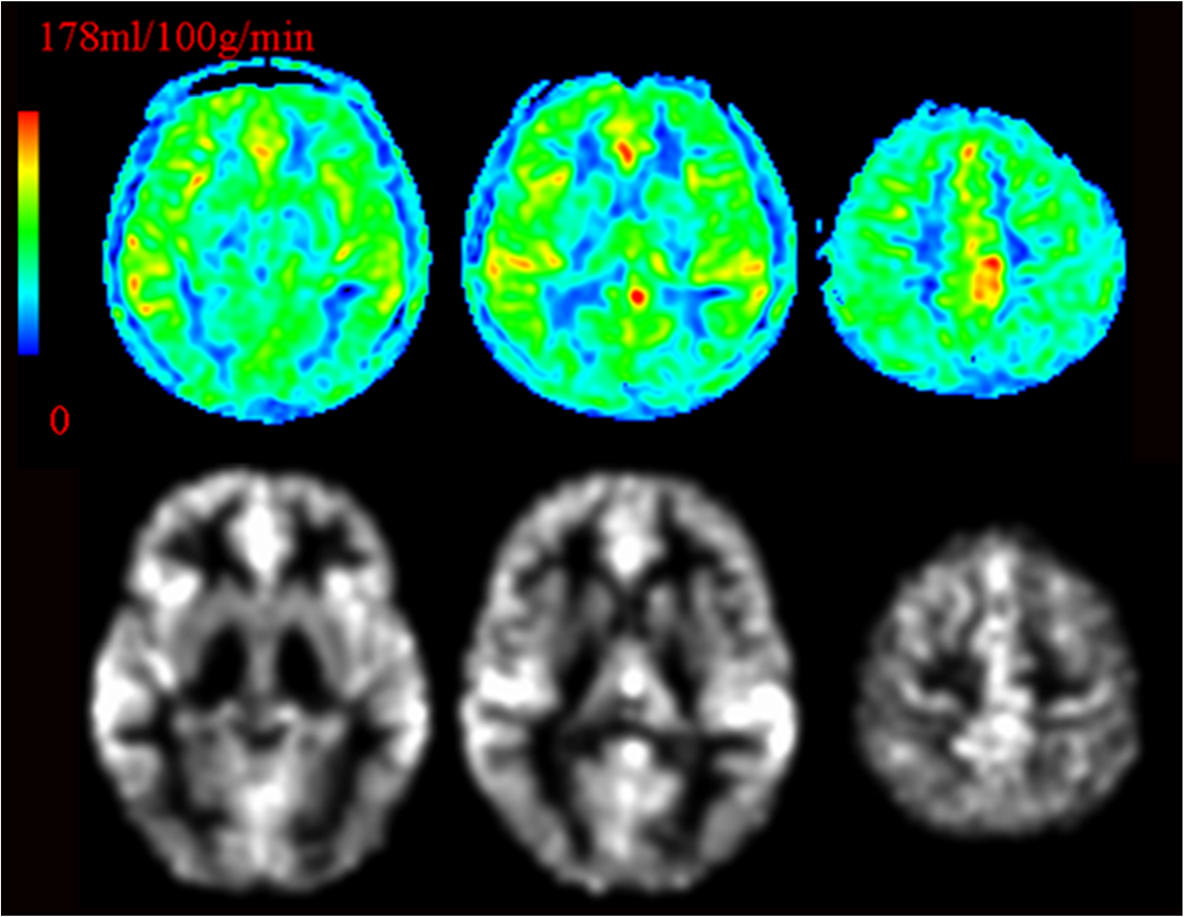
**Representative raw CBF images (first row) and spatially normalized smoothed CBF images after partial volume correction (nsCBF**_**correct**_**) from an adolescent with IGA (the second row).** Note: The left part of the figure represents the patient’s right side. IGA = Internet gaming addiction; CBF = cerebral blood flow.

Group differences among the spatially normalized and smoothed CBF_correct_ (nsCBF_correct_) maps in the IGA and control groups were tested by voxel-wise, two-sample t-tests. Multiple comparison correction was performed using the AlphaSim program in the Analysis of Functional Neuroimages software package, as determined by Monte Carlo simulations. Statistical maps of the two-sample *t*-test were created using a combined threshold of *p* < 0.05 and a minimum cluster size of 54 voxels, yielding a corrected threshold of *p* < 0.05. Regions exhibiting statistically significant differences were masked on MNI brain templates. The CIAS measures the severity of IGA, and previous studies show that patients with IGA have impaired impulse control [36]. A previous study has also reported a significant positive correlation between the severity of Internet addiction and the level of impulsivity [37]. To determine whether altered perfusion in subjects with IGA was related to the severity of IGA symptoms, impulsivity severity in IGA, or hours of Internet use per week, we calculated correlations between the mean nsCBF_correct_ values in all clusters that survived AlphaSim correction and CIAS scores, BIS-11 scores, and hours of Internet use per week in the 15 subjects with IGA. Because the nsCBF_correct_ image was generated from the raw CBF image by several post-processing steps, its value could not be expressed in absolute units of milliliters per 100 g per minute. Figure 1 showed the representative raw CBF images and spatially normalized smoothed CBF images after partial volume correction (nsCBF_correct_) from an adolescent with IGA. The normal CBF value of the brain parenchyma varied from 40 to 100 ml100 g^-1^ min^-1^[38], and our raw CBF map was consistent with it.

## Results

### Demographic and behavioral and personality measures

Table 1 and Table 2 list the demographic and behavioral and personality measures we collected in the IGA and control subjects. There were no significant differences in the distributions of age, gender, and years of education between the two groups. The subjects with IGA engaged in more hours of Internet use per week (p < 0.0001) and had higher CIAS (p < 0.0001) and BIS-11 (p = 0.02) scores than the control subjects. No differences in SAS or SDS scores were found between the groups.

**Table 1:** Demographic and behavioral characteristics of the included participants

	**Adolescent with**	**Control group**	**P value**
	**internet gaming**	**(n = 18)**	
	**addiction**		
	**group (n = 15)**		
	**(Mean ± SD)**	**(Mean ± SD)**	
Age (years)	16.93 ± 2.34	16.33 ± 2.61	0.50
Gender (M/F)	13/2	14/4	0.67
Education (years)	9.53 ± 2.17	9.56 ± 3.01	0.98
Hours of Internet use per week	25.47 ± 17.89	9.28 ± 12.90	<0.0001
Chen Internet Addiction Scale (CIAS)	66.73 ± 3.01	40.50 ± 8.42	<0.0001
Self-Rating Anxiety Scale (SAS)	44.33 ± 5.72	41.17 ± 4.45	0.08
Self-rating depression scale (SDS)	50.20 ± 7.55	50.06 ± 7.26	0.96
BIS-11 Attentional key	14.80 ± 2.65	13.06 ±2.34	0.05
Motor key	19.67 ± 3.90	18.11 ±2.70	0.18
Non-planning key	26.67 ± 3.15	23.94 ± 3.02	<0.0001
total score	61.13 ± 7.61	55.11 ± 5.92	0.02

*Abbreviation: SD* standard deviation, *CIAS* Chen Internet Addiction Scale, *SAS* Self-Rating Anxiety Scale, *SDS* Self-rating depression scale, *BIS*-*11* Barratt Impulsiveness Scale-11.

Two-sample *t* test was used for group comparisons but chi-square was used for gender comparison.

**Table 2:** Demographic data, CIAS, SAS, SDS, and BIS-11 scores, and hours of Internet use per week in all participants

	**Sex**	**Age**	**Eyr**	**Hours**	**CIAS**	**SAS**	**SDS**	**BIS**-**11**			
								**Attentional key**	**Motor key**	**Non-planning key**	**Total score**
IGA1	m	11	4	24	64	48	53	18	21	26	65
IGA2	m	16	9	25	66	40	48	13	12	28	53
IGA3	m	18	12	50	65	36	43	12	17	22	51
IGA4	m	19	12	21	66	50	50	15	19	31	65
IGA5	f	18	9	26	69	53	50	14	18	22	54
IGA6	m	19	9	20	64	51	46	19	30	30	79
IGA7	m	19	9	30	75	45	70	14	17	28	59
IGA8	m	17	9	17	70	45	54	13	19	27	59
IGA9	m	19	12	15	64	45	48	12	22	26	60
IGA10	m	19	11	19	67	44	54	14	19	25	58
IGA11	f	18	12	16	67	33	35	12	19	22	53
IGA12	m	14	7	14	66	43	45	15	20	24	59
IGA13	m	17	10	25	64	38	49	13	20	31	64
IGA14	m	15	9	20	65	50	55	19	18	29	66
IGA15	m	15	9	60	69	44	53	19	24	29	72
CON1	f	18	13	40	46	43	45	13	20	21	54
CON2	m	13	7	5	51	46	55	15	21	28	64
CON3	m	11	4	0	29	43	54	12	16	25	53
CON4	m	11	4	20	49	41	51	11	13	19	43
CON5	m	18	13	15	54	33	39	11	21	22	54
CON6	f	18	12	2	42	45	64	14	19	26	59
CON7	m	14	7	2	33	43	43	10	15	25	50
CON8	m	18	9	14	37	39	43	10	17	19	46
CON9	m	18	12	2	26	33	51	13	18	28	59
CON10	m	18	12	1	49	35	45	13	16	24	53
CON11	m	17	12	0	45	40	58	12	20	25	57
CON12	m	19	9	0	37	43	51	12	19	22	53
CON13	f	14	8	2	47	38	55	14	15	23	52
CON14	m	19	13	10	45	41	40	15	20	22	57
CON15	m	14	6	10	32	43	51	13	16	22	51
CON16	m	18	12	15	39	50	56	18	18	26	62
CON17	m	16	9	35	40	40	41	11	18	30	59
CON18	f	16	9	3	28	45	59	18	24	24	66

*Abbreviation: Eyr* Educational years, *Hours* Hours of Internet use per week, *SAS* Self-Rating Anxiety Scale, *CIAS* Chen Internet Addiction Scale, *SDS* Self-rating depression scale, *BIS*-*11* Barratt Impulsiveness Scale-11.

### Between-group analysis of CBF

**Figure 2:**
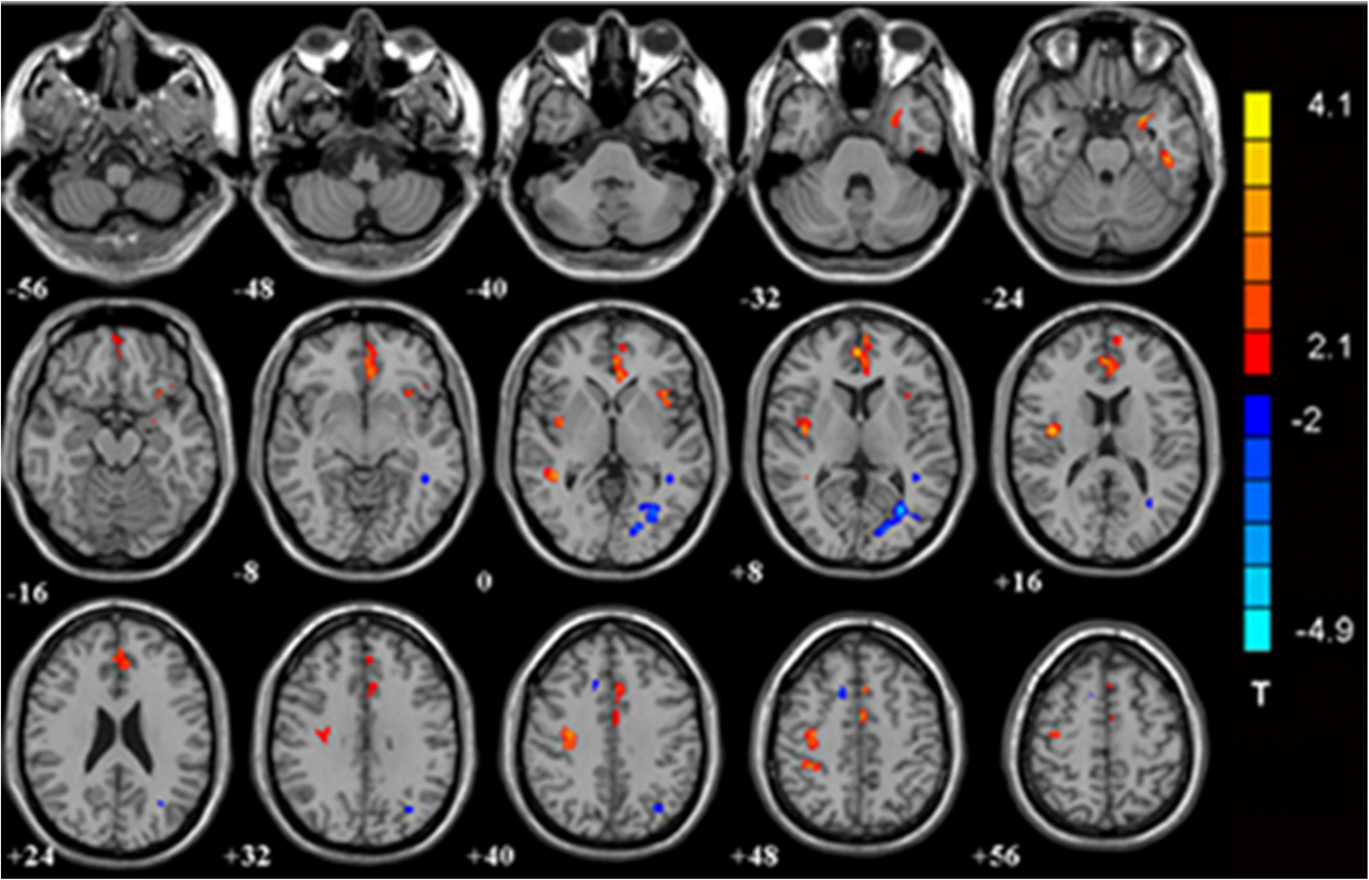
**Significant between-group differences in CBF between control subjects and those with IGA.** The IGA group showed increased CBF in the left inferior temporal lobe, left parahippocampal gyrus and amygdala, right medial frontal lobe/anterior cingulate cortex, bilateral insula, right middle temporal gyrus, right precentral gyrus, left supplementary motor area, left cingulate gyrus, and right inferior parietal lobe. Decreased CBF was found in the left middle temporal gyrus, left middle occipital gyrus, and right cingulate gyrus (*p* < 0.05, AlphaSim-corrected). The t-score bars are shown on the right. Red indicates IGA > controls and blue indicates IGA < controls. Note: The left part of the figure represents the patient’s right side. IGA = Internet gaming addiction; CBF = cerebral blood flow.

Compared with the control group, the IGA group showed increased CBF in the left inferior temporal lobe, left parahippocampal gyrus/amygdala, right medial frontal lobe/anterior cingulate cortex, bilateral insula, right middle temporal gyrus, right precentral gyrus, left supplementary motor area, left cingulate gyrus, and right inferior parietal lobe. Decreased CBF was found in the left middle temporal gyrus, left middle occipital gyrus, and right cingulate gyrus (Table 3, Figure 2).

**Table 3:** Summary of regional cerebral blood flow (CBF) changes in adolescents with IGA compared with normal controls

	**Peak MNI coordinate region**	**Peak MNI coordinates**			**Number of cluster voxels**	**Peak T value**
		**x**	**y**	**z**		
1	Left inferior temporal lobe/Fusiform gyrus	−44	−28	−24	75	3.38
2	Left parahippocampal gyrus/Amygdala	−26	0	−22	130	3.32
3	Right medial frontal lobe/Anterior cingulated cortex	2	50	6	867	4.12
4	Left insula	−38	18	2	168	3.23
5	Right insula	42	−8	14	214	3.76
6	Right middle temporal gyrus	42	−44	2	84	3.41
7	Right precentral gyrus	30	−16	38	357	3.49
8	Left supplementary motor area	−4	18	48	143	2.95
9	Left cingulated gyrus	−2	−2	50	85	3.24
10	Right inferior parietal lobe	52	−34	52	95	2.98
11	Left middle temporal gyrus	−44	−46	−4	89	−3.25
12	Left middle occipital gyrus	−30	−68	6	478	−4.93
13	Right cingulate gyrus	14	16	46	78	−2.97

*Abbreviation: MNI* Montreal Neurological Institute, *IGA* internet gaming addiction, *CBF* cerebral blood flow.

### Correlation between nsCBF_correct_ values and CIAS scores, BIS-11 scores, and hours of internet use per week in subjects with IGA

There were no significant correlations between mean nsCBF_correct_ values in any of the thirteen regions listed in Table 3 and CIAS or BIS-11 scores or hours of Internet use per week.

## Discussion

The non-invasive nature of ASL has opened a unique window into human brain function and perfusion physiology. High spatial and temporal resolution makes this technique very appealing, for both the diagnosis of vascular diseases and in basic neuroscience studies where the aim is to develop a more comprehensive picture of the physiological events accompanying neuronal activation [38]. The overall goal of ASL is to produce a flow-sensitized image or “labelled” image and a “control” image in which the static tissue signals are identical, but where the magnetization of the inflowing blood differs. The subtraction control- label yields a signal difference, △*M*, that directly reflects local perfusion because the signal from stationary tissue is completely eliminated. The Label is usually performed by inverting or saturating the water molecules of the blood supplying the imaged region. By adding a delay between the labelling and image acquisition, called inversion delay, the labelled blood spins are allowed to reach the capillaries, where they exchange with tissue water and thereby give rise to the perfusion signal [38]. The two main classes of ASL are continuous ASL (CASL) and pulsed ASL (PASL). The advantages of ASL—including independence from injected contrast agents, lack of irradiation, and fast acquisition times—may facilitate intensive perfusion studies of the early recognition of schizophrenia and other psychiatric disorders, and longitudinal disease-monitoring research of these conditions. With continuing improvements in the precision of MRI-based perfusion techniques, it is increasingly feasible to use this tool in the study of the subtle brain perfusion changes occurring in psychiatric disorders and substance abuse research [39]. The ability to measure CBF is very important for the assessment of tissue metabolism and function. The non-invasiveness of ASL, which allows pro-longed functional studies to be performed, makes it a preferable choice in many neuroscience applications. Following a neuronal activation paradigm, the blood oxygen level dependent (BOLD) signal [40] is a result of T_2_* changes that are a consequence of altered CBF and cerebral blood volume, and the cerebral metabolic rate of oxygen uptake. The ASL signal, however, is an absolute measure of CBF changes, which makes this technique more reproducible over time, both within and between subjects [41]. In addition, perfusion functional MRI is believed to localize regions of activation more accurately [42] than BOLD imaging, which is affected by changes in deoxygenated blood in draining venous vessels resulting in additional signal from “downstream” areas [38].

In the past decade, research has accumulated suggesting that excessive Internet use can lead to the development of a behavioral addiction. Internet addiction has been considered as a serious threat to mental health and the excessive use of the Internet has been linked to a variety of negative psychosocial consequences [43]. Behavioral addictions such as IGA are similar to drug addiction except that the term “addiction” is now being applied to a range of excessive behaviors, such as gambling, video game playing, eating disorders and other behaviors [44]. On the molecular level, IGA is characterized by an overall reward deficiency that entails decreased dopaminergic activity. On the level of neural circuitry, Internet and gaming addictions lead to neuroadaptations and structural changes as a consequence of prolonged high levels of activity in brain areas associated with addiction. On a behavioral level, Internet and gaming addicts present constricted cognitive functioning in various domains [43]. Research indicates that brain activity alterations commonly associated with substance-related addictions occur following the compulsive engagement in behaviors, such as pathological gambling [45]. In line with this, we propose that similar mechanisms and changes are involved in IGA.

Previous PET and single photon emission computed tomography (SPECT) studies focused on examining the reward circuitry through dopamine-transporter metrics rather than CBF in Internet addicts. They reported reduced dopamine transporter availability in Internet addicts [46], especially in the bilateral dorsal caudate and right putamen [47]. Koepp et al. provided the first evidence of striatal dopamine release during video game play, which was similar to that following amphetamine or methylphenidate injections [48]. The authors reported that Internet addiction was related to neurobiological abnormalities in the dopaminergic system that were similar to those found in substance-related addictions.

In the scientific literature, there are only a few studies that have investigated CBF and metabolism changes in adolescents with IGA. Park et al. [17] investigated differences in regional cerebral glucose metabolism during the resting state between young individuals with IGA and normal controls using ^18^ F-fluorodeoxyglucose PET (^18^ F-FDG PET) study [17]. Their results showed that the IGA group had increased glucose metabolism in the right middle orbitofrontal gyrus, the left caudate nucleus, and the right insula and decreased metabolism in the bilateral postcentral gyrus, the left precentral gyrus, and the bilateral occipital regions compared with normal users. They suggested that Internet game overuse may be associated with abnormal neurobiological mechanisms in the orbitofrontal cortex, striatum, and sensory regions, which are implicated in impulse control, reward processing, and somatic representation of previous experiences. Their results also supported the idea that Internet game overuse shares psychological and neural mechanisms with other types of impulse control disorders and substance/non-substance-related addiction [17]. Ha-Kyu et al. performed ^18^ F-FDG PET in six normal control subjects and eight IGA patients comparing rest and 20 minutes of engaging in each subject’s favorite Internet game [16]. They found that the IGA group showed more areas of activation in the frontal lobe and parietal lobe than the control group. This suggests that addictive use of Internet games may result in functional alteration of the developing brain in adolescents.

We found that the IGA group showed increased CBF in the left inferior temporal lobe, left parahippocampal gyrus and amygdala, right medial frontal lobe/anterior cingulate cortex, bilateral insula, right middle temporal gyrus, right precentral gyrus, left supplementary motor area, left cingulate gyrus, and right inferior parietal lobe compared with the control group. Most of these areas were mentioned in a model proposed by Volkow et al. [9]. They presented a model according to which addiction emerges as an imbalance in information processing and integration among various brain circuits and functions. These dysfunctions reflect (a) decreased sensitivity of reward circuits, (b) enhanced sensitivity of memory circuits to conditioned expectations to drugs and drug cues, stress reactivity, (c) negative mood, and (d) a weakened control circuit [9]. They described a number of neurobiological systems that may mediate cue-induced gaming craving. These include visual processing areas such as the occipital lobe or precuneus that link gaming cues to internal information, and memory systems that include the hippocampus, parahippocampus, or amygdala, which provide emotional memories and contextual information for the gaming cues. They also include reward systems such as the limbic system and posterior cingulate that allow for the evaluation of gaming-related information and provide expectations and reward significance, and they include motivation systems such as the anterior cingulate and orbital frontal lobe that control the desire for gaming. Finally, these systems include executive systems such as the dorsolateral prefrontal cortex and prefrontal cortex that allow one to form a plan to get online for gaming.

Our results are in line with the model proposed by Volkow et al. [9]. The amygdala and hippocampus are part of a circuit involved in learning and memory that has been associated with intense craving in response to drug-associated cues [49]. The insula is known to play a crucial part in addiction because of its role in conscious urges to abuse drugs [50]. Functional imaging studies have shown activation of the insula during urges for cigarette smoking [51], cocaine [52], and alcohol [53], and insular activity has frequently correlated with self-reported urges [51,53]. The prefrontal cortex is thought to be an important area with respect to drug-seeking behavior and craving [54,55]. The activity of the pre-frontal cortex, particularly the anterior cingulate cortex, has been positively correlated with craving for alcohol, cocaine, marijuana, and tobacco during cue presentation [51,56-58]. Han et al. [59] reported that brain activity in the anterior cingulate and orbitofrontal cortex increased among the excessive Internet game playing group following exposure to Internet video game cues relative to general players. They also reported that increased craving for Internet video games correlated with increased activity in the anterior cingulate for all participants. This indicated craving for online games alters brain activity irrespective of addiction status and may therefore be seen as a (prodromal) symptom of addiction, and addicted players can be distinguished from non-addicted online gamers by a different form of brain activation. It has been consistently reported that the parietal cortex is activated by a response inhibition task, and some researchers have hypothesized that the parietal cortex may play a role in regulating attention or withholding motor responses during response inhibition tasks [60,61]. Some researchers [62] have suggested that parietal overactivity is either an underlying cause of poor inhibition or a response to failures of inhibition. Recently, a functional MRI study revealed cue-induced activation of the right insula in subjects with IGA [63].

Decreased CBF was found in the left middle temporal gyrus, left middle occipital gyrus, and right cingulate gyrus. Temporal gyrus and occipital gyrus are thought to be responsible for visual and auditory functions, and Dong suggested that the gaming process requires players to pay full attention to each subtle change in the screen and endure the noisy sounds of the game for a long time. Stimulation of visual and auditory related brain regions for a long time may have made them insensitive to excitation. Sustained over stimulation of visual attention can impair subjects’ visual functions and noise can impair their hearing abilities [64,65]. As such, sustained game playing may impair a player’s visual and auditory abilities. Our observed pattern of hyper-perfusion is not totally in agreement with the findings in several resting-state perfusion studies of substance addiction. It was interesting that we detected increased CBF in the left cingulate gyrus but decreased CBF in the right side, and this should be investigated in future studies.

It is difficult to determine whether hyper-perfusion phenomenon is a compensatory response or is directly induced by the addictive behavior itself. Rao et al. used continuous ASL perfusion MRI to measure the effects of *in utero* cocaine exposure (IUCE) on resting brain function by comparing resting CBF in 24 cocaine-exposed adolescents with 25 control subjects [66]. They found cocaine-exposed adolescents showed significantly reduced global CBF primarily in posterior and inferior brain regions, including the occipital cortex and thalamus. After adjusting for global CBF, however, a significant increase in relative cerebral blood flow was found in the prefrontal, cingulate, insular, amygdala, and superior parietal cortex in cocaine-exposed adolescents. These frontal, cingulate, and parietal regions serve as the neural substrates mediating attention and arousal regulation. The changes in relative CBF in these regions are consistent with the view that changes induced by IUCE affect attention processing [67,68]. Gottschalk and Kosten examined 20 control subjects and 32 recently abstinent cocaine abusers using SPECT [69]. They found that CBF abnormalities varied by brain location, with hypo-perfusion significantly more likely in occipital and temporal cortex or cerebellum and hyper-perfusion more likely in frontal and parietal cortex. Up to now, it has remained unclear whether these CBF increases reflected primarily neurological lesions or secondary changes to compensate for such damage, and the investigators hypothesized that increased brain blood flow reflected a compensatory response to decreases in CBF [69].

### Limitations

There are several limitations that should be mentioned in regard to this study. First, although ASL-MRI is noninvasive and entails no exposure to ionizing radiation, intravenous contrast agents, or radioactive isotopes, its sensitivity is still limited for routine clinical applications because of a low intrinsic perfusion signal/noise ratio compared with other methods, such as dynamic contrast enhancement MRI, ^18^ F-FDG PET, and 99mTc–hexamethylpropyleneamine oxime (HMPAO) SPECT. This can affect precise quantification of perfusion, affect the perfusion status of individuals, and compromise statistical validity. However, in its current version, it is impossible to derive an estimate of the spread of the data within each group and the amount of overlap between groups. Such detail at the individual level is important because a clinical diagnostic technique will have to be applicable in the individual patient [32]. Second, we applied partial volume correction for CBF using segmented tissue maps from high resolution 3D T1-weighted images. The results of the partial volume correction should depend on the segmentation results. Further methodological advances will likely improve this correction technique. Third, the sample size was relatively small, which can reduce the power of the statistical analyses and hamper generalization of the findings. As such, the reported results should be considered preliminary, and they should be replicated in future studies with larger sample sizes. Fourth, as a cross-sectional study, our results do not clearly demonstrate whether the psychological features preceded the development of IGA or were a consequence of the overuse of the Internet. Finally, we could not exclude potential residual confounding effects such as levels of physical activity and school performance. Therefore, future prospective studies should clarify the causal relations between IGA and psychological measures.

## Conclusions

We used 3D ASL perfusion fMRI and noninvasively quantified resting CBF in adolescent participants with and without IGA, demonstrating that IGA alters CBF distributions in the adolescent brain. These results support the hypothesis that IGA is a behavioral addiction that may share similar neurobiological abnormalities with other addictive disorders. Although this is a preliminary study, perfusion fMRI may be a valuable tool for imaging the effects of sustained internet game play in adolescents.

## Competing interests

The authors do not have an affiliation with or financial interest in any organization that might pose a conflict of interest.

## Authors’ contributions

Authors YZ, JS, JX and YD conceived and designed the experiments. Authors YS, XC and ZZ performed the experiments. Authors YZ, YS, WD analyzed the data. Authors QF, XC, YZ and JX wrote the paper. All authors read and approved the final manuscript.
